# The role of patient expectations in predicting outcome after total knee arthroplasty

**DOI:** 10.1186/ar2811

**Published:** 2009-09-21

**Authors:** Anne F Mannion, Stephane Kämpfen, Urs Munzinger, Ines Kramers-de Quervain

**Affiliations:** 1 Department of Research and Development, Schulthess Klinik, Lengghalde 2, 8008 Zürich, Switzerland; 2 Department of Rheumatology and Institute of Physical Medicine, University Hospital Zürich, Gloriastrasse 25, 8091 Zürich, Switzerland; 3 Department of Lower Extremity Orthopaedic Surgery, Schulthess Klinik, Lengghalde 2, 8008 Zürich, Switzerland; 4 Department of Rheumatology, Schulthess Klinik, Lengghalde 2, 8008 Zürich, Switzerland

## Abstract

**Introduction:**

Patient's expectations are variably reported to influence self-rated outcome and satisfaction after medical treatment; this prospective study examined which of the following was the most important unique determinant of global outcome/satisfaction after total knee arthroplasty (TKA): baseline expectations; fulfilment of expectations; or current symptoms and function.

**Methods:**

One hundred and twelve patients with osteoarthritis of the knee (age, 67 ± 9 years) completed a questionnaire about their expectations regarding months until full recovery, pain, and limitations in everyday activities after TKA surgery. Two years postoperatively, they were asked what the reality was for each of these domains, and rated the global outcome and satisfaction with surgery. Multivariable regression analyses using forward conditional selection of variables (and controlling for age, gender, other joint problems) identified the most significant determinants of outcome.

**Results:**

Patients significantly underestimated the time for full recovery (expected 4.7 ± 2.8 months, recalled actual time, 6.1 ± 3.7 months; *P *= 0.005). They were also overly optimistic about the likelihood of being pain-free (85% expected it, 43% were; *P *< 0.05) and of not being limited in usual activities (52% expected it, 20% were; *P *< 0.05). Global outcomes were 46.2% excellent, 41.3% good, 10.6% fair and 1.9% poor. In multivariable regression, expectations did not make a significant unique contribution to explaining the variance in outcome/satisfaction; together with other joint problems, knee pain and function at 2 years postoperation predicted global outcome, and knee pain at 2 years predicted satisfaction.

**Conclusions:**

In this group, preoperative expectations of TKA surgery were overly optimistic. The routine analysis of patient-orientated outcomes in practice should assist the surgeon to convey more realistic expectations to the patient during the preoperative consultation. In multivariable regression, expectations did not predict global outcome/satisfaction; the most important determinants were other joint problems and the patient's pain and functional status 2 years postoperatively.

## Introduction

It is now generally accepted that the outcome of total joint replacement should be assessed not only on the basis of imaging, technical results, and objective functional/physiological findings, but also in relation to the patient's perception of the benefit gained, as regards domains of importance to them in their everyday life [1,2]. Patients' expectations of treatment are a potentially important determinant of their subsequent ratings of outcome, yet one that remains relatively unexplored in the fields of rheumatology and orthopaedic surgery [3]. Various theoretical models exist describing the relationship between expectations and satisfaction in the setting of medical care. The most dominant model posits that expectations being met - that is, minimising the mismatch between prior expectations and the actual result - is the most important determinant [4,5]. Other models, however, maintain that higher expectations *per se *are associated with better outcomes [6,7], perhaps reflecting the influence of dispositional optimism [8] or a sort of placebo effect. Further models suggest that the actual post-treatment status with regards to symptoms or function more strongly governs whether the patient is satisfied with the results, regardless of their prior expectations [9,10].

Only few studies have examined the relationship between expectations and outcome/satisfaction in relation to total joint replacement surgery, and even fewer specifically in relation to the knee joint. Engel and colleagues examined the influence of baseline expectations regarding improvement in the condition and regarding change in quality of life on outcome [11], as measured with both disease-specific and generic instruments, 6 months after total knee arthroplasty (TKA). They revealed that these expectations accounted for between 9% and 13% of the variance in outcome, depending on the instrument used. They did not, however, investigate how well the expectations met the reality of the situation at follow-up, or whether this had any independent influence on outcome ratings or satisfaction.

Mahomed and colleagues examined the importance of expectations (dichotomised as high or low, with respect to expected changes in pain, functional limitations, overall success, and the likelihood of complications) in predicting outcomes after total joint arthroplasty [12]. In multivariable analyses, expectations about pain (but not any other domains) had significant predictive value with respect to the outcomes of Western Ontario and McMaster Universities Osteoarthritis Index pain, of Western Ontario and McMaster Universities Osteoarthritis Index function, and of Short Form-36 function, although the unique variance accounted for in each case was relatively low. These authors, too, did not examine how well the reality of the outcome had met the prior expectations of the patients, or whether this influenced their satisfaction with treatment.

Burton and colleagues *did *examine the notion of expectations being met in relation to the outcome of total joint replacement (hip) [13], and noted that expectations were fulfilled in just over one-half (55%) of the patients interviewed. A high proportion of patients (86%) nonetheless claimed that the operation had been successful - although the unfulfilled patients reported a significantly lower quality of life than those whose expectations were met [13]. Unfortunately, the investigation was retrospective, with patients being required to recall their preoperative expectations of an average 3.5 years ago; it is well known that the data collected using such study designs are subject to strong recall bias and potential confounding by the actual outcome of the surgery. The study by Mancuso and colleagues of total hip replacement patients was beset with the same limitations of the retrospective study design; these authors also reported a high proportion of satisfied patients overall (89%), but satisfaction rates were lower in those expecting improvement in nonessential activities (perhaps suggesting overly ambitious or unrealistic expectations) and those with a poor postoperative condition [14].

Moran and colleagues [15] quantified the preoperative expectations of hip and knee total joint replacement patients, by asking them to rate their current status on the Oxford hip or Oxford knee questionnaires and to predict the level of symptoms expected 6 months after surgery. The operating surgeons also completed the latter task. It was shown that the surgeons expected significantly better results than the patient. The researchers, however, did not go on to examine these expectations in relation to the actual changes achieved or the patients' satisfaction with their postoperative status.

In summary, previous investigations in the field of joint replacement have delivered inconclusive findings, in part due to the retrospective nature of the investigations or failure to use multivariable models to identify the relative importance of putative predictors.

The present study seeks to expand our knowledge of the relationship between expectations and outcome, measured as satisfaction with surgery and the global outcome of surgery, in patients undergoing joint replacement for osteoarthritis of the knee. Specifically, in multiple regression analysis we tested, when controlling for potential confounding variables, which (if any) of the following variables made a unique significant contribution to explaining the variance in satisfaction and in global outcome 2 years after TKA: baseline expectations, the actual knee status (pain and function) at 2-year follow-up, and expectations being fulfilled (preoperative declared expectations minus 2 year postoperative actual status).

## Materials and methods

### Overview of the study

The patients described in the present investigation were participating in a large-scale prospective study examining subjective and objective aspects of locomotor function before and after TKA (results on objective changes in function to be reported elsewhere). The participants completed questionnaires before total joint replacement surgery and again 2 years later. The study group comprised those with questionnaire data from both baseline and follow-up assessments (n = 112/146 (77%); for details on drop-outs, see later).

The patients received an oral and written explanation of what would be required of them, and signed an informed consent form confirming their agreement to participate. The study was approved by the local University Ethics Committee.

### Study admission criteria

All patients who were scheduled for a primary knee arthroplasty (TKA) at the authors' hospital in the year of study were invited to participate; approximately 55% volunteered. The only inclusion criteria were a willingness to comply with the test battery and complete the follow-up assessments, and a good understanding of written German. No patients were excluded on the basis of their age or activity level.

### Questionnaires

#### Pre surgery

Approximately 2 weeks before the operation, during a visit to the hospital for the accompanying functional tests (reported elsewhere), the patients completed the Total Arthroplasty Outcome Evaluation Questionnaire Baseline and History Forms of Katz and colleagues [16] (modified for the knee; the actual questionnaire can be found in the Appendix of Katz and colleagues [16]). The Baseline form enquired, amongst other things, about the patient's main *reasons for choosing *knee replacement surgery (10 options - multiple answers allowed, with the most important reason also to be indicated); the importance of decreasing pain and increasing function; and *expectations of surgery *in relation to expected time until full recovery (open answer, in months), expected pain after recovery from surgery (not at all painful through to very painful), and expected limitations in everyday activities after recovery from surgery (not limited at all through to greatly limited).

The History Form enquired (amongst other things) about various sociodemographic characteristics, pain in the left and right knees (recoded to obtain the answer for the index knee - four categories: no pain, slight pain, moderate pain, severe pain), and extent of limitation in usual activities (five categories: not limited at all, slightly limited, moderately limited, greatly limited, totally limited).

The form also enquired about the involvement of other joints by asking 'Other than your knee, what areas are very painful?' (none, back and/or buttocks, left hip, right hip, other - give details). The answer was then dichotomised as yes if any of the joints given in the option list (or feet as other) were indicated, and as no if the answer was none or any other areas of the body, with the rationale that these other joints might affect overall mobility/locomotor function.

The American Society of Anesthesiologists Physical Status Classification System was used to assess the patient's overall physical health (1 = normal healthy, 2 = mild/moderate systemic disease, 3 = severe systemic disease, 4 = life-threatening systemic disease), since it was considered that this may have influenced the patient's function or postoperative outcome.

#### Two years post surgery

Two years after surgery, when the patients attended for their follow-up assessment, they completed the same items from the History Form to assess *current status *in relation to the domains that had been enquired about in the preoperative expectations questionnaire (months required until recovered, pain, limitations in everyday activities). They also completed the Post-operative Form, which asked them to rate the *global outcome/result of surgery *(1 = excellent, 2 = good, 3 = fair, 4 = poor) and their *satisfaction with surgery *(1 = very satisfied, 2 = somewhat satisfied, 3 = somewhat dissatisfied, 4 = very dissatisfied) - these two measures were to serve as the dependent variables in the multiple regression analyses - and to state whether they would choose to undergo the procedure again if they found themselves in the same situation, knowing what they now know about the outcome (yes, definitely; yes, probably; no, probably not; no, definitely not).

In summary, *expectations *were measured at baseline, and current *pain and function *were measured prospectively (each preoperatively and at 2 years), also yielding a measure of the *change in pain and function*. In each expectations domain (time to recovery, pain, and function), the difference between the preoperative expected score and the follow-up actual score yielded a measure of the extent to which *expectations had been fulfilled*.

### Statistical analysis

Descriptive data are presented as the mean and standard deviations unless otherwise stated. Contingency analyses were used to examine associations between categorical variables. Bivariate analyses (Spearman rank or Pearson correlations, as appropriate) were used to examine the zero-order correlations between global outcome (or satisfaction) and the potential predictors.

Multiple linear regression analyses were carried out to identify the variables that made a significant unique contribution to explaining the variance in outcome, using firstly global treatment outcome and then satisfaction with treatment as the dependent variable to be predicted. Age, gender, and presence of other joint problems were entered into the model as a first step, to control for these potential confounding variables. After this, the following variables were entered using a forward conditional selection criterion (with a probability-of-F-to-enter ≤ 0.05): the two expectations items (that is, regarding expected pain and function); pain and function at 2 years; the change in pain and the change in function (in each case, the value measured prospectively from pre surgery to 2-year follow-up); and the fulfilment of expectations (expectations minus actuality) scores for each of the three domains.

Collinearity was assessed by examining the tolerance values and variance inflation values for the independent variables in the final regression models; values < 0.1 and > 5, respectively, were considered to suggest problematic collinearity [17] (no problems with collinearity were found within the analyses carried out).

Statistical analyses were carried out using Statview (SAS Institute Inc, San Francisco, CA, USA) and SPSS version 16.0 for Apple Macintosh (Chicago, IL, USA).

Statistical significance was accepted at the *P *< 0.05 level and no corrections were made for multiple testing [18].

## Results

The baseline sociodemographic and pain/function data for the group of 112 patients with questionnaire data at baseline and at 2-year follow-up are presented in Table 1. The 34 drop-outs showed a nonsignificant tendency to be older (70 ± 9 years) than the patients who completed the 2-year follow-up (67 ± 9 years) (*P *= 0.07), but showed no significant differences from those with 2-year data regarding gender distribution (*P *= 0.70), body weight (*P *= 0.99), height (*P *= 0.56), baseline pain (*P *= 0.86) and baseline functional limitations (*P *= 0.36). The reasons for dropping out were that seven patients had died, one patient had moved abroad, four patients had undergone revision and did not want to continue, five patients had other operations or physical problems, one patient did not go on to operation (heart problem), and 16 simply did not want to continue with the study. Of the 34 drop-outs, 17 patients had actually returned for a clinical check-up with the physician at 2 years: review of the medical notes indicated that 13 of these patients had no pain, two patients had pain, and two patients had no specific information on pain; 11 patients were satisfied with the results of the operation, one patient was dissatisfied, and five patients had no specific information; and 13 patients had good function, one patient had poor function, and three patients had no specific information on function.

**Table 1:** Baseline sociodemographic, pain, function and co-morbidity characteristics of patients

Variable	Baseline value
Demographic/physical variables	
Gender (*n*)	78 women, 34 men
Age (years)	67 ± 9
Body weight (kg)	87.5 ± 15 (men), 76.6 ± 15.5 (women)
Job status (%)	
Full time	14
Part time	9
Retired/unemployed/homemaker	77
Marital status (%)	
Married	61
Widowed	20
Divorced/separated	12
Never married	7
Living conditions (%)	
Alone	29
With partner	65
With family	6
Pain, function and co-morbidity variables	
Affected knee (%)	
Left	46
Right	48
Both within 3 months	6
Pain duration (%)	
< 6 months	1
6 to 12 months	9
1 to 3 years	23
3 to 5 years	14
> 5 years	53
Pain intensity (%) (n = 110)	
None	1
Slight	3
Moderate	30
Severe	66
When is the pain bothersome? (%)	
Have no pain	1
First few steps only	6
After long walks (> 30 minutes)	10
Whenever walk	43
Constantly, even at rest	40
Knee limits ability to do sports (%)	
No limitation	0
Slightly limits me	0
Moderately limits me	6
Greatly limits me	28
Totally limits me	30
Do not participate in sport for reasons unrelated to my knee	35
Knee limits/interferes with sexual activity (%)	
No limitation	24
Slightly limits me	11
Moderately limits me	16
Greatly limits me	8
Totally limits me	1
Not sexually active for reasons unrelated to my knee	40
Knee limits ability to work (%)	
No limitation	8
Slightly limits me	13
Moderately limits me	22
Greatly limits me	21
Totally limits me	6
Not working for reasons unrelated to my knee	30
Other painful joints (back, hip, foot) (%)	65
American Association of Anaesthesiologists co-morbidity grade (%)	
Grade I	17
Grade II	51
Grade III	32

Data presented as *n*, mean ± standard deviation, or percentage.

Thirteen out of the 112 patients with baseline data and 2-year follow-up data had undergone some sort of further surgery on the same knee, between 1 month and 21 months after the index surgery (eight early wound revisions, including evacuation of haematomas; four revisions with exchange of the implant; and one secondary implantation of a patella component). As expected, this group recorded significantly worse 2-year global outcomes (*P *= 0.003) and satisfaction grades (*P *= 0.012) than the rest of the group - since these revisions could not have been anticipated at baseline, yet they may have had an influence on the overall outcome rating at 2 years, the data from this group were not included in the multivariable analyses of predictors of outcome.

### Reasons for surgery

By far the most common primary reason for deciding to undergo TKA, given by over one-half of those responding (53/99, 53.5%), was 'I can't stand the pain any longer; something has to be done'. This was followed by 'I want to walk without a limp, and/or without using a cane/crutch' (17.2%), 'I want to increase my walking endurance' (14.1%), and 'doctor's recommendation' (6.1%). The other six options were each chosen by 1 to 3% patients (13 patients were not able to answer the question).

The distribution of answers (n = 111) to the question 'In deciding to have knee replacement surgery, how important was it for you to decrease your pain' was as follows: 44.1% extremely important, 51.4% very important, 3.6% moderately important, and just 0.9% slightly important. The same question in relation to 'increasing your ability to do normal activities' returned the following answer distribution (n = 112): 48.2% extremely important, 47.3% very important, and 4.5% moderately important.

### Preoperative expectations regarding recalled time to recovery

**Figure 1:**
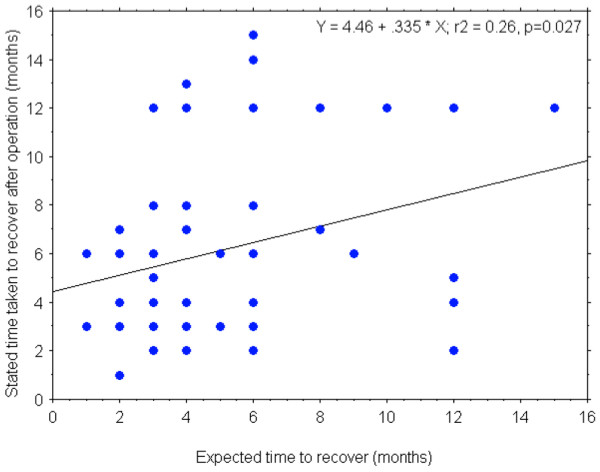
Time for recovery from total knee arthroplasty. Relationship between the expected time required to recover from the total knee arthroplasty and the actual time required to recover, as recalled 2 years postoperatively.

The expected mean time until recovery was 4.7 ± 2.8 months; in reality, by the 2-year follow-up only 80% of the patients actually considered themselves fully recovered from the operation, and they recalled that it had taken them, on average, 6.1 ± 3.7 months to do so. Figure 1 shows a scatter plot of the individual values for the expected time to recovery and the recalled time taken to recover after the TKA. Although the correlation between the two variables was significant, the absolute agreement was poor in many cases.

### Preoperative expectations compared with actual results 2 years after surgery

The preoperative expectations for pain and function compared with the actual outcome at 2 years follow-up are presented in Table 2.

**Table 2:** Distribution of baseline expectations and actual status at 2-year follow-up for pain and for function

Expected status, declared pre operation	Actual status 2 years post operation				
How painful?	Not at all	Slightly	Moderately	Very	All options
	
Not at all	*45 (43)*	**32 (30)**	**12 (8)**	**5 (4)**	94 (85)
Slightly	3 (2)	*4 (4)*	**7 (4)**	**3 (3)**	17 (13)
Moderately	-	-	-	-	-
Very	-	-	-	-	-
All options	48 (45)	36 (34)	19 (12)	8 (7)	111^a ^(98)
					
How limited in function?	Not at all	Slightly	Moderately	Greatly	All options
	
Not at all	*16 (16)*	**22 (21)**	**15 (10)**	**5 (3)**	58 (50)
Slightly	5 (5)	*12 (12)*	**18 (17)**	**13 (9)**	48 (43)
Moderately	0	0	0	**4 (4)**	4 (4)
Greatly	0	0	1 (1)	0	1 (1)
All options	21	34	34	22	111^a^

Values in parentheses are those for the group excluding patients who had undergone further operations on the knee. Pain - in the whole group, expectations were met in 44% of patients (values in italics), were not met in 53% (values marked bold), and were exceeded in 3%. Function - in the whole group, expectations were met in 25% of patients (values in italics), were not met in 70% (values marked bold), and were exceeded in 5%. ^a^One patient had missing data preoperatively, hence n = 111(*98*).

Consistent with the most common reason for deciding to undergo surgery, the majority of patients (94/111, 85%) declared that they expected no knee pain and the remainder (17/111, 15%) declared expectation of only slight knee pain after surgery. In reality, only 43% of the group reported being pain-free at 2 years post operation. The patients were similarly overly optimistic about function, with the majority of the group expecting no limitations (58/111, 52%) or only slight limitations (48/111, 43%) after surgery, but with only 20% and 30% patients, respectively, achieving such a status.

On an individual basis, expectations regarding pain were met or exceeded in 47% patients; for function, just 30% achieved their expected function or better (Table 2).

### Global outcome and satisfaction 24 months post surgery

The ratings of the global outcome of the knee replacement 24 months after surgery (n = 112) were as follows: 46.4% excellent, 42.0% good, 9.8% fair, and 1.8% poor (excluding the revision patients, ratings were 49.5%, 41.4%, 9.1% and 0%, respectively).

The ratings for satisfaction with the results of the knee replacement (n = 112) were similarly distributed, although with somewhat more patients in the highest category: 58.6% very satisfied, 31.5% satisfied, 8.1% somewhat dissatisfied, and 1.8% dissatisfied (excluding the revision patients, ratings were 62.3%, 29.6%, 7.1% and 1.0%, respectively).

### Decision to undergo surgery

In response to the question 'Now that you have learned a lot about knee replacement surgery, if you could go back in time and make the decision again, would you choose to have the surgery?', 73.9% patients said 'yes, definitely', 18.9% said 'yes, probably', 6.3% said 'no, probably not', and 0.9% said 'no, definitely not'.

### Interrelationships between the baseline and outcome variables

Table 3 presents the bivariate correlations between the various predictors (baseline demographics and clinical status, baseline expectations, pain/function at 2 years post operation, change in pain from pre operation to 2 years post operation, and fulfilment of expectations) and global outcome and satisfaction.

**Table 3:** Correlation matrix showing inter-relationships between the examined predictors, global outcome and satisfaction

	Gender (male 0, female 1)	Age	Other joint problems (no 1, yes 2)	ASA score (co-morbidity)	**Pain pre operation**^a^	**Functional limitations pre operation**^a^	**Expectations about pain**^a^	**Expectations about functional limitations**^a^	Expectations about recovery time	**Pain at 2 years**^a^	**Functional limitations at 2 years**^a^	**Change in pain, pre operation to 2 years**^a^	**Change in functional limitations, pre operation to 2 years**^a^	**Expectations fulfilled, pain**^b^	**Expectations fulfilled, functional limitations**^b^	**Global treatment outcome**^c^
Gender (male 0, female 1)	1.000															
Age	0.073	1.000														
Other joint problems (no 1, yes 2)	-0.089	-0.163	1.000													
ASA score (co-morbidity)	0.025	0.388**	0.224*	1.000												
Pain pre operation^a^	0.021	0.055	-0.111	0.010	1.000											
Functional limitations pre operation^a^	0.044	-0.020	-0.010	-0.169	0.218	1.000										
Expectations about pain^a^	0.000	-0.240*	0.077	-0.026	0.019	0.096	1.000									
Expectations about functional limitations^a^	-0.271*	-0.004	0.146	0.176	0.117	0.010	0.255*	1.000								
Expectations about recovery time	-0.014	-0.292**	0.179	-0.181	0.062	-0.100	0.136	0.030	1.000							
Pain at 2 years^a^	-0.053	-0.288**	0.231*	0.089	0.221*	0.055	0.312**	0.303**	0.175	1.000						
Functional limitations at 2 years^a^	-0.131	-0.143	0.163	0.110	0.349**	0.267*	0.342**	0.492**	0.020	0.389**	1.000					
Change in pain, pre operation to 2 years^b^	0.035	0.335**	-0.288**	-0.048	0.420**	0.083	-0.318**	-0.198	-0.171	-0.765**	-0.158	1.000				
Change in functional limitations, pre to 2y^b^	0.154	0.133	-0.138	-0.204	-0.231*	0.344**	-0.293**	-0.469**	-0.073	-0.325**	-0.791**	0.171	1.000			
Expectations fulfilled, pain^b^	0.045	0.226*	-0.236*	-0.091	-0.253*	-0.017	0.023	-0.225*	-0.140	-0.930**	-0.291**	0.678**	0.248*	1.000		
Expectations fulfilled, functional limitations^b^	-0.044	0.146	-0.090	-0.052	-0.326**	-0.256*	-0.183	0.059	-0.009	-0.261*	-0.818**	0.065	0.618**	0.230*	1.000	
Global treatment outcome^c^	0.007	-0.111	0.302**	0.032	0.041	0.085	0.236*	0.237*	0.150	0.405**	0.384**	-0.389**	-0.262*	-0.355**	-0.255*	1.000
Satisfaction^c^	-0.014	-0.154	0.323**	0.100	0.010	0.036	0.274*	0.262*	0.102	0.567**	0.264*	-0.543**	-0.194	-0.498**	-0.094	0.800**

Data in bold are significant: *P < 0.05 (two-tailed), **P < 0.01 (two-tailed). n = 80 patients (listwise exclusion of missing data, and excluding patients (n = 13) that underwent further surgery on the index knee). ASA, American Association of Anaesthesiologists. ^a^Higher value = more pain (or expect more pain) or greater functional limitations (or expect greater functional limitations). ^b^Higher value = better outcome (greater change in pain or functional limitations, greater fulfilment of expectations). ^c^Higher value = worse outcome, or lower satisfaction.

### Expectations, change in symptoms, and the expectations- actuality discrepancy as predictors of global rating of outcome

The results of the final step of the multiple regression analyses are presented in Tables 4 and 5. In predicting the global treatment outcome, the simultaneous entry of the control variables at the first step (demographic and baseline clinical variables) explained a significant proportion of the variance (*P *= 0.034); the variable having other joint problems made a unique significant contribution, also in the final model (*P *= 0.046). At the second step, knee pain at the 2-year follow-up was selected for entry, with a significant 20.5% increase in the step change in *R*^2 ^(*P *< 0.0001). At the third step, functional limitations at the 2-year follow-up explained a further significant 4.4% variance (*P *= 0.022). In the final model, the variables that made a significant unique contribution were other joint problems, knee pain at 2 years, and knee functional limitations at 2 years - with higher values for each being associated with a poorer global outcome.

**Table 4:** Results of multiple regression analysis explaining variance in global outcome at 2 years

Step	Step change in *R*^2^	*P *value for step change in *R*^2^	β for final model (only significant predictor variables shown)	*P *value
First	0.098	0.034	0.189 (other joint problems)	0.046
Second	0.205	< 0.0001	0.384 (pain at 2 years)	0.001
Third	0.044	0.022	0.238 (functional limitations at 2 years)	0.022
Adjusted *R*^2 ^for model	0.307			

Results of multiple regression analysis showing the factors that made a unique significant contribution to explaining variance in global outcome at 2 years (1 = excellent, 4 = poor). In the final model, the significant predictors of a poorer outcome were: other joint problems, more pain at 2 years post operation, and greater functional limitation at 2 years post operation. n = 87 patients (listwise exclusion of missing data, and excluding patients (n = 13) that underwent further surgery on the index knee). Apart from the demographic variables, predictor variables were entered on the basis of the significance of their bivariate correlation with the dependent variable: step 1, simultaneous entry for age, gender, other joint problems (yes/no); steps 2 and 3, forward conditional entry for preoperative expectations (about pain and about functional limitations), knee status at 2-year follow-up (in terms of pain and functional limitations), change in knee status from pre surgery to 2 years (in terms of pain and functional limitations), expectations - actuality scores for knee status (that is, expected status minus actual status at 2 years) (in terms of pain and functional limitations). Step change in *R*^2^, increase in explained variance at the given step; adjusted *R*^2^, *R*^2 ^- (*k *- 1)/(*n *- *k*) × (1 - *R*^2^), where *n *is the number of observations and *k *is the number of independent variables; β for final model, β value after all variables have been entered; *P *value, significance of final β value for the stated variable.

**Table 5:** Results of multiple regression analysis explaining variance in satisfaction at 2 years

Step	Step change in *R*^2^	*P *value for step change in *R*^2^	β for final model (only significant predictor variables shown)	*P *value
First	0.095	0.040	0.194 (other joint problems)	0.042
Second	0.231	< 0.0001	0.517 (pain at 2 years)	< 0.0001
Adjusted *R*^2 ^for model	0.293			

Results of the multiple regression analysis showing the factors that made a unique significant contribution to explaining the variance in satisfaction at 2 years (1 = very satisfied, 4 = very dissatisfied). In the final model, the significant predictors of a poorer outcome were: other joint problems, more pain at 2 years post operation, and greater functional limitation at 2 years post operation. n = 87 patients (listwise exclusion of missing data, and excluding patients (n = 13) that underwent further surgery on the index knee). Apart from the demographic variables, predictor variables were entered on the basis of the significance of their bivariate correlation with the dependent variable: step 1, simultaneous entry for age, gender, other joint problems (yes/no); step 2, forward conditional entry for preoperative expectations (about pain and about functional limitations), knee status at 2-year follow-up (in terms of pain and functional limitations), change in knee status from pre surgery to 2 years (in terms of pain and functional limitations), expectations - actuality scores for knee status (that is, expected status minus actual status at 2 years) (in terms of pain and functional limitations). Step change in *R*^2^, increase in explained variance at the given step; adjusted *R*^2^, *R*^2 ^- (*k *- 1)/(*n *- *k*) × (1 - *R*^2^), where *n *is the number of observations and *k *is the number of independent variables; β for final model, β value after all variables have been entered; *P *value, significance of final β value for the stated variable.

A similar pattern of variable selection was seen when satisfaction with treatment was used as the dependent variable, although in the final model only other joint problems (*P *= 0.042) and knee pain at 2 years (*P *< 0.0001) were unique significant predictors (model *R*^2 ^= 29%; Table 5).

Although relevant in the bivariate analyses, in neither of the multivariable models did baseline expectations or expectations being fulfilled make a significant contribution to explaining the variance in global outcome or satisfaction, when the 2-year status for pain and for functional limitations were also included in the model.

## Discussion

The present study sought to examine the extent to which patient self-ratings of global outcome and satisfaction after TKA were determined by prior expectations of the outcome, by expectations being met, or by the actual symptom/functional status after surgery. Studies supporting each of these putative predictors of satisfaction have been reported in the literature in relation to the treatment of various medical conditions [3,4,6].

Overall, the results did not support the notion that expectations *per se *are important unique determinants of outcome: the results showed low but significant associations with global outcome and satisfaction in bivariate analyses (Table 3), but in the multivariable analyses they did not explain any additional variance in outcome once the (more significant) current pain/functional status variables had been selected for entry. Some previous studies in orthopaedics also found no unique role for expectations *per se *in predicting the improvement in function [19] or the global outcome of surgery [20]. Other authors found that baseline expectations in some domains explained up to 13% of the variance in total joint replacement outcome [11,12], measured using either generic, joint-specific or pain-scale instruments. In neither of these studies, however, was the relationship between expectations and *global outcome *or *satisfaction *assessed. Also in the present study, bivariate analyses showed that baseline expectations predicted the change in pain and change in functional limitations, accounting for a similar proportion of variance to that reported by Engel and colleagues [11] and by Mahomed and colleagues [12] (9 to 16%, *r *= 0.3 to 0.4; Table 3); however, these results did not retain significance in the multivariable model predicting the overall global outcome or satisfaction. De Groot and colleagues reported that spine surgery patients who had optimistic expectations about postoperative pain were less disappointed with surgery than were patients with pessimistic expectations, although the same did not apply for the outcomes rate of recovery or return to work [21]. Further, similar to the results of the multivariable analysis in the present study, it transpired that when the postoperative back pain at 3 months was considered a covariate in predicting disappointment with surgery, the influence of baseline expectations regarding pain was lost [21]. It therefore appears that the actual status may be more predictive than expectations *per se *when satisfaction or global outcome is modelled using multivariable techniques.

In the present study, in both of the multivariable regression models, the most significant predictor of the 2-year global outcome/satisfaction was the current knee status (pain and functional limitations). Interestingly, and in contrast to some previous studies [4,5,20], the variable describing the fulfilment of expectations for pain (expectations- actuality discrepancy) did not achieve significance in the multivariable model, even though it had shown a significant correlation with both global outcome and satisfaction in the bivariate analyses (*r *= 0.3 to 0.5, *P *< 0.05). This was most probably the result of the high correlations between pain/functional limitations at the 2-year follow-up and the fulfilment of expectations in these domains (*r *= 0.8 to 0.9; Table 3), leading to just one of these two variables retaining significance in the given multivariable model.

The patients' expectations of surgery declared in the present investigation were quite high, and were overly optimistic compared with the actual results achieved. The vast majority (85%) of patients expected to be pain free, yet only 43% were; and 52% expected to have no functional limitations, yet just 20% achieved this. This overestimation of the probable improvement after TKA [12,13] and other kinds of elective orthopaedic surgery [20,22] has been reported before. Mahomed and colleagues found that, in a mixed sample of hip and knee arthroplasty patients, 75% expected to be pain-free and 40% expected to be unlimited in their usual activities [12]. Burton and colleagues reported that the majority expected to be pain-free but only 55% actually were [13]. In most expectations studies, the present one included, it is not known whether expectations reflect dispositional optimism (that is, the expectation that good outcomes generally occur when confronted with problems across important life domains) [8] or reflect considered expectations based on information received (for example, during the consultation, through patient information sources, personal experience), or indeed a combination of both. Either way, these findings in relation to the overestimation of the probable result of surgery highlight the importance of both routine outcome assessment and longitudinal studies of the factors influencing outcome, to guide informed discussion with the patient regarding the extent of improvement that can realistically be achieved.

The negative influence of other joint problems on the probable outcome of TKA may need to be emphasised to a greater extent in the preoperative informed consent process. As banal as it may seem, it is important that patients with co-morbidity in terms of other joint problems (though according to the present study not in relation to general co-morbidity as measured with the American Association of Anaesthesiologists co-morbidity score) are made aware that the operation is being carried out for the specific knee joint disease identified, and that it will not necessarily serve as a general panacea for other ongoing medical problems. Indeed, ongoing pain and functional limitations in connection with other joint problems will probably persist after the surgery, and influence general functioning and the quality of life accordingly. If this is not explicitly discussed with the patient prior to surgery, then inappropriate expectations may go unchecked, ultimately leading to disappointment with the result.

The salient features of the present study include its prospective nature, its relatively large sample size, its examination of different domains for which the patient may hold expectations, and its multivariable approach to the analysis. Further, the overall proportion of successful outcomes (88.5% excellent and good) was similar to the figures presented in previous studies (86% [13], 85% [23]), providing confidence in the generalisability of the findings. Several limitations, however, must also be acknowledged.

The questionnaire used to assess the (preoperative) expectations of improvement and the (postoperative) achievement of improvement and overall outcome has not been validated for use in the knee; it was originally developed and validated for use in the hip [16]. Many of the current hip and knee questionnaires, however, show considerable overlap in their item content (for example, the Oxford hip questionnaire and the Oxford knee questionnaire [24]), and the items in the Total Arthroplasty Outcome Evaluation Questionnaire appeared to display acceptable face validity also for the knee. In fact, no questions in the questionnaire relate to specifically hip-related activities and most questions just focus on pain and general activities that affect the lower extremities. Nonetheless, future studies would be required to establish the questionnaire's construct validity by comparison with other knee-specific questionnaires. Furthermore, our German version of the Total Arthroplasty Outcome Evaluation Questionnaire did not undergo the currently recommended, stringent procedure for the cross-cultural adaptation of questionnaires [25]; indeed, the questionnaire was produced for use in the hospital before the widespread adoption of such guidelines. Primarily, it represented a translation by a bilingual (first language, German) rheumatologist, cross-checked by a bilingual colleague (first language, English) and reviewed by various bilingual clinicians.

The Total Arthroplasty Outcome Evaluation Questionnaire was chosen for use in the present investigation because, when the study was first designed, this instrument appeared to offer one of the most comprehensive, but simple and efficient, means of assessing the many domains/constructs of interest in arthroplasty patients. Most of the individual scales in the questionnaire are single-item measures (that is, one item per construct) with adjectival or Likert scales; although a number of studies have shown that these can be just as valid and representative of a domain as multi-item scales [26,27], it would be of interest to confirm the present findings using the currently more popular multi-item scales such as the Oxford-12, the Western Ontario and McMaster Universities Osteoarthritis Index, and so forth [28]. Similarly the use of a more extensive questionnaire to assess co-morbidity might deliver more precise information about other illnesses/disorders potentially influencing outcome [29] than does the American Association of Anaesthesiologists co-morbidity score.

In relation to the statistical analyses used in the present study, the regression models examined the main effects of level of expectations, regardless of whether these were met, and of fulfilled expectations, independent of their preoperative level. However, perhaps the interaction of both should be considered, for example to investigate whether expectations that are met lead to a good outcome only when expectations are high. This particular analysis could not be carried out in the present study, because, for the given sample size, the power using moderated hierarchical regression analysis would have been too low [30] and because there were so few patients with low expectations that the moderated test would have been somewhat biased. Future studies should address these issues.

## Conclusions

In the patient group examined, patient expectations of surgery were generally overly optimistic. This highlights the importance of routinely assessing patient-orientated outcome and the various factors influencing it, such that realistic expectations for different outcome domains can be discussed with the individual patient prior to surgery. Although in bivariate analyses expectations being met were significantly associated with outcome, in the final multivariable model only the presence of other joint problems and the degree of improvement in symptoms and function were unique significant determinants of a good global outcome and of satisfaction with the procedure.

## Abbreviations

TKA: total knee arthroplasty.

## Competing interests

The authors declare that they have no competing interests.

## Authors' contributions

IK-dQ and UM were responsible for the conception and design of the main study, of which this substudy is part, and acquired funding for the project; they also coordinated all of the practical work and acquisition of data. AFM performed the statistical analysis, interpreted the data and drafted the manuscript. SK organised and prepared the data for analysis, and assisted with some of the statistical analyses and with the writing of the manuscript. All authors read and approved the final manuscript.
